# Cytokines as an important player in the context of CAR-T cell therapy for cancer: Their role in tumor immunomodulation, manufacture, and clinical implications

**DOI:** 10.3389/fimmu.2022.947648

**Published:** 2022-09-12

**Authors:** Caio Raony Farina Silveira, Amanda Cristina Corveloni, Sâmia Rigotto Caruso, Nathália Araújo Macêdo, Natália Moscheta Brussolo, Felipe Haddad, Taisa Risque Fernandes, Pamela Viani de Andrade, Maristela Delgado Orellana, Renato Luiz Guerino-Cunha

**Affiliations:** ^1^ Advanced Cellular Therapy Laboratory, Fundação Hemocentro de Ribeirão Preto, São Paulo, Brazil; ^2^ Cell Therapy Laboratory, Fundação Hemocentro de Ribeirão Preto, São Paulo, Brazil; ^3^ Department of Medical Images, Hematology and Clinical Oncology, Ribeirão Preto Medical School of University of São Paulo, Ribeirão Preto, Brazil

**Keywords:** CAR-T cells, chimeric antigen receptor, cytokines, immunomodulation, cell therapy, cancer microenvironment, immunotherapy

## Abstract

CAR-T cell therapies have been recognized as one of the most advanced and efficient strategies to treat patients with hematologic malignancies. However, similar results have not been observed for the treatment of solid tumors. One of the explanations is the fact that tumors have extremely hostile microenvironments for the infiltration and effector activity of T-cells, mainly due to the presence of highly suppressive cytokines, hypoxia, and reactive oxygen species. Taking advantage of cytokines functionally, new fourth-generation CAR constructs have been developed to target tumor cells and additionally release cytokines that can contribute to the cytotoxicity of T-cells. The manufacturing process, including the use of cytokines in the expansion and differentiation of T cells, is also discussed. Finally, the clinical aspects and the influence of cytokines on the clinical condition of patients, such as cytokine release syndrome, who receive treatment with CAR-T cells are addressed. Therefore, this review aims to highlight how important cytokines are as one of the major players of cell therapy.

## Introduction

CAR-T cell therapy has recently emerged as a promising treatment for hematological malignancies (1, 2). Its effectiveness depends on an entire formation process, from construct engineering and manufacturing to clinical response. During these steps, cytokines perform distinct roles that directly impact the efficiency of this therapeutic resource once they are closed related to the regulation of inflammatory pathways. In addition, cytokines have an important contribution to the development of the suppressor tumor microenvironment (TME) given opportunities to design new strategies and alternatives for TME immunomodulation. For this purpose, new CAR vectors of fourth generation have being designed to incorporate, in the addition of CAR construct, other genes able to express pro-inflammatory cytokines that might favor the efficacy of this therapies (2, 3).

In the manufacturing process of CAR-T cells, cytokines have a key role in the activation, expansion of immune cells and in the selection/polarization in subpopulations, such as memory T cells. Memory T cells potentially might impact patient outcome due to their superior persistence, resistance to apoptosis, and inferior predisposition to the development of exhaustion (4).

After the infusion of CAR-T cells, patients potentially experiment a cytokines storm condition, called as Cytokine Release Syndrome (CRS) (5). In terms of clinical response, despite the outstanding performance of CAR-T cells for hematological neoplasm treatment, their successful application in solid tumors still presents several challenges. The selection of potential tumor-specific target antigens and tumor stroma provide a physical barrier to the CAR-T cells infiltration to the TME (2, 6), in addition to the immunosuppressive TME, a well-known down-regulating mechanism of the immune cells antitumor response (7). Among the mechanisms of immunosuppression and tumor immunoescape are the secretion of regulatory cytokines, recruitment and polarization of regulatory T cells, myeloid suppressor cells (e.g., TAM, TAN, MDSC), and expression of immune checkpoints that lead to T-cell anergy and depletion of essential amino acids for the proliferation of specific lymphocytes (8, 9).

This review aims to critically discuss the cytokines as an important player in the context of a highly complex scenario of CAR-T cell therapy for cancer focused on discussing the implications of cytokines functionalities in the design and engineering of new CAR constructs, manufacturing protocols and in the clinical response.

## The role of cytokines in the maintenance of the immunosuppressive phenotype and the impact on the CAR-T cells function

Tumors are complex tissues that contain, in addition to tumor cells, endothelial cells, pericytes, inflammatory cells, fibroblasts, as well as acellular components, such as cytokines, lipid mediators and extracellular matrix. Within the TME, different elements are capable of inducing angiogenesis, promote the survival and proliferation of tumor cells and inactivate T cells antitumor responses through various mechanisms (10).

Tumor-infiltrating inflammatory cells are attracted to TME up cytokines secreted by tumor cells (11). In the first instance, they constitute an important initial line of defense against the tumor, through the generation of reactive oxygen species, secretion of pro-inflammatory cytokines, and mediating cytotoxicity. However, this pro-inflammatory environment is counterbalanced with the development of a chronic inflammation into the TME, leading the local tissue microenvironment to change into a growth-promoting state (12, 13). Cytokines have a prominent role in this scenario, once they are able to modulate the antitumor responses, cell transformation and malignancy proliferation due to the chronic inflammation (14).

Despite being a genetically modified T cells, CAR-T cells undergo the same process of turning off responses as non-genetically modified cells. Taking advantage of cytokines function, the TME cells impact negatively the proliferation, differentiation, activation, migration and survival of immunological cells which constitute a hostile microenvironment; variables closely correlated with the biology of immunological CAR-T cell therapy (15).

## IL-2 and TGF-β

Some cytokines induce patterns response pro-tumor such as TGF-β, secreted by two major types of immunosuppressive cells of the TME, the myeloid-derived suppressor cells (MDSCs) and regulatory T cells (Tregs) (16).

Based on the TGF-β activity, the differentiation of Tregs (CD25^+^Foxp3^+^) impact the response of T cells and the neutralization of their activities is strongly implicated with the anti-tumor immune response (17). Tregs limit the expansion of effector T cells in the TME by capturing IL-2, as they have a high affinity trimeric receptor to this cytokine (7). Moreover, *in vitro* experiments showed that TGF-β suppressed the proliferation of human T cells through inhibition of the IL-2 receptor (18) and it reduces the production of IL-2 itself by inhibiting the activity of the IL-2 promoter. *In vivo*, TGF-β promoted downregulation of CD8^+^ and CD4^+^ T cell expansion (19) and cytotoxic T cells activity by several mechanisms, such as inhibition of the expression of perforin, granzyme and INF-y (20, 21). In mice, the deficiency of the production of TGF-β1 and TGF-β receptor in T cells, correlated with greater proliferation and activation of T cells subtypes helper 1 (Th1), as well as the production of Th2 cytokines (22, 23).

The close relationship of TGF-β with the IL-2 has important manufacturing and clinical implications, once most strategies for CAR-T cell processing are the IL-2 based manufacturing protocols. IL-2 is an important cytokine that can promote a relatively mature phenotype, with low expression of CD62L, CCR7, CD27 and CD28, which correlated with a reduced blood persistence *in vivo* and terminal effector T cells differentiation and proliferation (19).

To overcome the action of TGF cytokine’s immunosuppressive actions of the TME, the use of mesothelin-targeted chimeric antigen receptor T cell therapy together with an oncolytic adenovirus (rAd.sT) expressing sTGFβRIIFc (soluble transforming growth factor beta receptor II-Fc fusion protein) demonstrated enhanced anti-tumor responses against breast cancer tumor cells (24). Besides, Kloss and colleagues generated dominant-negative TGF-βRII-modified CAR-T cells specific for prostate-specific membrane antigen (PSMA), that exhibited superior level of proliferation, cytokine secretion, resistance to dysfunctionality related to chronic inflammation, long-term *in vivo* persistence, and the induction of tumor eradication in aggressive human prostate cancer mouse models (25).

Other studies have shown that the inhibition of the endogenous TGF-β receptor (TGFBR2) signal in CAR-T cells enhanced the anti-tumor function of ROR1-specific CAR-T cells against triple-negative breast cancer by reducing Treg conversion and also prevented the exhaustion of CAR-T cells (26, 27).

Potential strategies to enhance clinical response of CAR-T cell therapy might be targeting TME cells involved in the immunosuppression or combined cytokines production that could positively impact antitumor responses of immune cells, including the genetic modified ones, such as CAR-T cells.

## IL-4

IL-4 is involved in the differentiation of naive CD4 T cells. It can favor tumor growth by inhibiting tumor-directed Th1-polarized effector and inducing Th2 cells, which have been associated with less antitumor activity than IFN-ℽ producing CD4 T cells (Th1). Additionally, several cancer types express IL-4 and the IL-4R, which suggests a role in tumor progression cancer (28).

However, modified cytokine receptors, including dominant negative receptor (DNR) and inverted cytokine receptor (ICR), can redirect this immunosuppressive cytokine in the TME towards pro-inflammatory response. Base on the knowledge that IL-7 is implicated with the proliferation and diversity of effector T cells in the TME with consequent superior antitumor effect, some strategies have been to merge the IL-7 receptor endodomain to the IL-4 receptor exodomain. This approach might be able, in the clinics, to promote a positive effect on cell expansion by the action of the IL-7 receptor endodomain after the recognition of IL-4 present in the TME (29).

## IFN- ℽ, a dual cytokine

IFN-**ℽ** is a potent proinflammatory agent that plays an important role in cellular immunity and anti-tumor responses due to its cytostatic, antiproliferative, and pro-apoptotic effect on tumor cells, in addition to stimulating pro-inflammatory macrophages and inducing apoptosis of Tregs (30). The main sources of IFN-**ℽ** are innate immunity cells, NK and NKT cells, and adaptive immunity cells, CD4 and CD8 T cells (10). Despite all the afore mentioned antitumor effects, IFN-**ℽ** can contribute to the tumor’s escape from immune surveillance. It has been reported that in some tumors, such as NSCLC (31) and ovarian cancer (32), IFN-**ℽ** can induce the expression of PD-L1 and IDO. Models of hepatoma, mammary adenocarcinoma and melanoma showed that chronic exposure to low doses of IFN-**ℽ** leads to tumor growth and induces the expression of molecules, such as PD-L1, PD-L2, CTLA-4 and Foxp3 (33), which are implicated to the dysfunction of T cell activity. Another way that IFN-**ℽ** can contribute to tumor progression is by generating genetic instability in cancer cells, which could lead to the alteration of tumor antigens and immune escape (34). Taking advantage of this knowledge, Zhang et al. (31) demonstrated that the IL-6/IFN-γ double knockdown CAR-T cells reduce the release of multiple cytokines from PBMCs *in vitro*, showing a promise strategy to be translated to the clinics.

## IL-6

Interleukin 6 is described as an important cytokine in tumor initiation and progression. Many studies reveal that high concentrations of IL-6 in the serum of patients with different types of cancer (such as gastric, melanoma, colorectal, pancreatic, among others) is related to poor prognosis of patients (35). The primary sources of IL-6 are tumor cells, MDSC, TAM, fibroblasts and CD8^+^ T cells. The effects of IL-6 can be direct on tumor cells, as well as indirectly through the promotion of angiogenesis and the modulation of stromal cells (36). Directly, IL-6 binds to its receptor on tumor cells and activates the STAT3 signaling pathway, which will lead to tumor progression through cyclin D1 and the proto-oncogene c-myc, an important regulator of the progression of G1 to S phase of the cell cycle (37). On the your side, STAT3 induces the anti-apoptotic proteins bcl-2, bcl-XL and survivin (38), which contribute to the survival of tumor cells. IL-6 also promotes the angiogenesis and metastasis that contribute to the expression of matrix metalloproteinase 2 (MMP-2) (39), expression of VEGF and bFGF (40) by tumor cells and also favor metastasis events by attracting metastatic cells outside the primary site of the tumor (41).

An interesting *in vivo* study demonstrated that ssCART-19 cells with *shRNA-IL-6* gene knockdown resulted in increased interferon-gamma (IFN-γ), tumor necrosis factor (TNF), interleukin-2 (IL-2), and IL-17A and decreased IL-10 and IL-6 levels. ssCART-19 inhibited the proliferation of Raji-Luc cells in tumor-bearing NSG mice, and reduced the incidence of lymphomas in the liver, kidneys, and spleen. The ssCART-19 DNA was mainly concentrated in the liver within 3 hours, and was widely distributed in most of the organs/tissues for 4 weeks after administration, demonstrating that ssCART-19 with *shRNA-IL-6* gene knockdown prolongs the survival time of tumor-bearing mice with a potential inferior risk of immunotoxicity and tumorigenicity (42).

**Figure 1:**
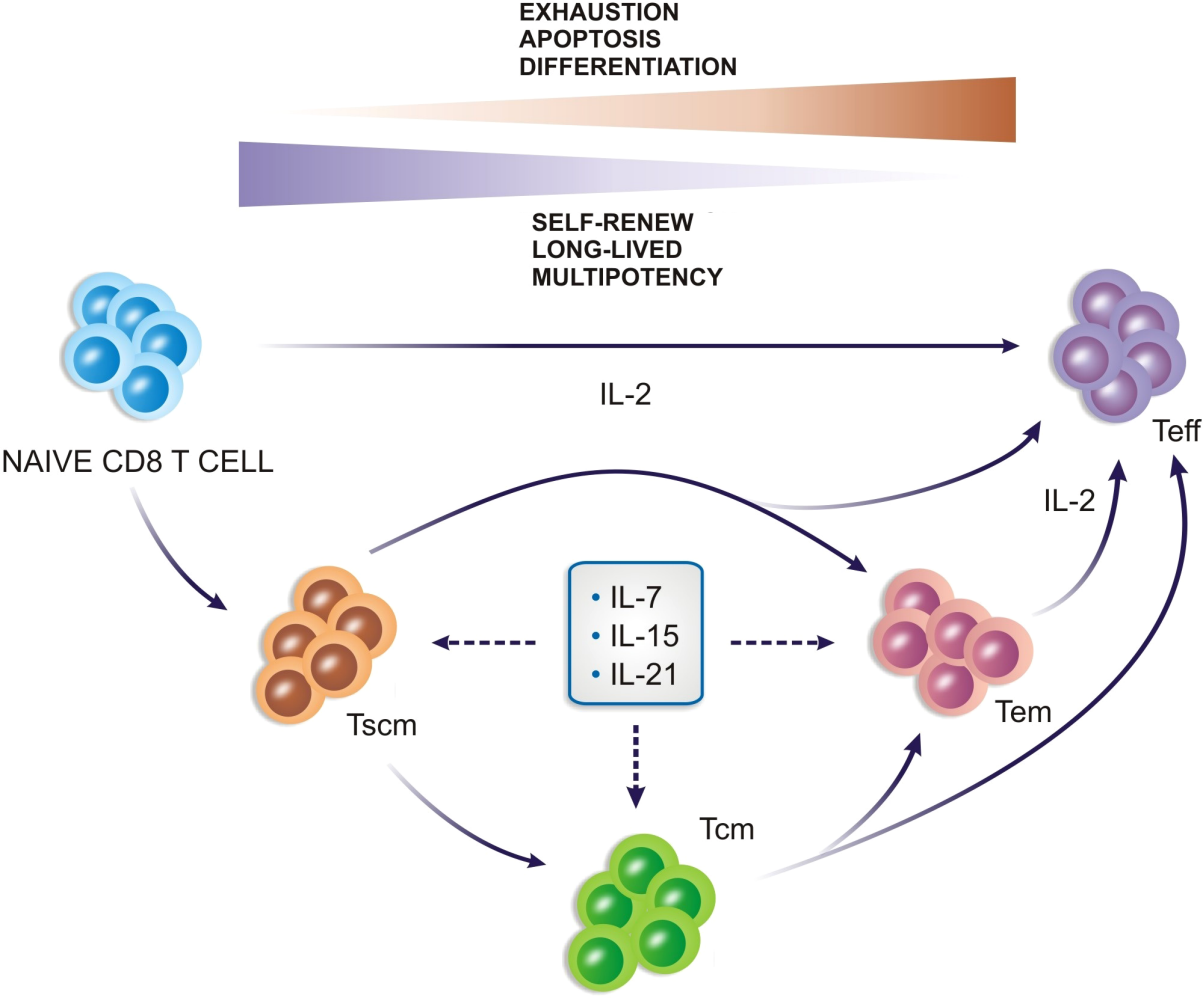
The cytokines dynamics in the tumor microenvironment and their role in the maintenance of the immunosuppressive phenotype. The microenvironment becomes hostile to the proliferation and effector functions of T cells and CAR-T cells.

It becomes increasingly important to better understand and take advantage of cytokines in the tumor’s biogenesis to modify the hostile TME and improve the therapeutic effect of CAR-T cells. In **Figure 1** it is shown the cytokines dynamics in the hostile TME.

## T-cells redirected for universal cytokine killing

Given the importance of cytokines in the biology of immunological therapies, including CAR-T cells, numerous studies have looked to modulate the profile of cytokines intended to enhance the effectiveness of these cells against diverse types of tumors (43–45).

Recently, a new generation of CAR T-cells was developed, “T-cells redirected for antigen-unrestricted cytokine-initiated killing” (TRUCKs), with the aim to associate the cytotoxicity of CAR T-cells with the delivery *in situ* of cytokines, aiming to circumvent the tumor microenvironment and modulating the immune system (43–46).

These CAR-T cells are modified with an (inducible) cytokine production cassette, such that the release of the cytokine is conditioned to the recognition of the antigen by the T cell and its activation (3, 47). This process requires the transfer of two transgenes (CAR and cytokine), which require integration into different genomic sites to avoid transactivation of the inducible cassette by the constitutive CAR promoter (3, 48).

Initially designed by Chmielewski et al., the study used the carcinoembryonic antigen (CEA)-specific CAR with the additional expression of IL-12, *in vitro* and *in vivo* models, demonstrated that IL-12-secreting CAR-T cells produced a greater pro-inflammatory response, improving T cell activation, modulated the tumor’s immune and vascular environment, and recruited additional immune cells, such as antitumor macrophages to the tumor microenvironment, significantly improving the antitumor response (46).

Previously, some approaches were tested with the aim to use IL-12 in the clinical setting. The use of IL-12 was implicated with systemic toxicity events and not feasible in some types of metastatic tumors. Alternatively, the to deliver IL-12 locally to the tumor lesion was also explored with no encourages results (1, 2, 6, 7). Compared with those strategies, CAR and iIL-12–modified T cells have the advantage to deliver IL-12 in a targeted tissue, and with satisfactory results, demonstrating to be a very promising approach (46).

In ovarian cancer which has a high immunosuppressive tumor environment (8, 49), the use of Muc16ecto specific CAR T cells modified to secrete IL-12 against ovarian cancer cells demonstrated by Koneru and colleagues (50) greater proliferation and increased IFN-γ secretion compared to CAR-T cells without IL-12. *In vivo*, the same study showed higher survival, prolonged persistence, and superior systemic IFN-γ, demonstrating greater antitumor efficacy of these cells (50). Subsequently, the same group extended their previous work using a syngeneic model of murine ovarian peritoneal carcinomatosis to characterize the mechanisms of these IL-12 secreting T CAR cells. Their data suggested that secreting CAR-T cells mediated tumor-associated macrophage depletion and resisted the inhibition induced by endogenous PD-L1, demonstrating that CAR IL-12 T cells were able to overcome these barriers by exercising a more efficient response. In addition, they have also demonstrated a potential acceptable profile of the toxicity of this therapy (50).

Currently, a panel of cytokines including IL-12, IL-7, IL-15, IL-18, IL-22, and IL-23 are being investigated. The IL-18 investigation came up with interesting results. Previous studies have shown that, in some models, higher levels of IL-18, in the absence of Th1 cytokines, promoted tumor progression (51) favored the occurrence of metastasis, and suppressed the function of NK cells by positive regulation of PD-1 (52, 53). On the other hand, other studies demonstrate that IL-18 supports an anti-tumor response of Tc1 cells by positive regulation of T-bet and suppression of Tc2 cells (54, 55). Recently, analyzing refractory pancreatic and lung tumors, Chmielewski and colleagues (56) demonstrated that IL-18 can polarize CAR T cells towards T-bethighFoxO^low^ effectors producing an acute inflammatory response. These cells (CAR-T/IL-18) induced a general change in the profile of immune cells associated with the tumor, increasing the number of CD206-M1 macrophages and NKG2D+ NK cells, and decreasing Tregs, suppressive CD103+ DCs, and M2 macrophages, besides prevented exhaustion of CAR T cells in the long term. They conclude that the association of IL-18 with CAR T cells are promisor strategy for the treatment of advanced solid cancer, as these cells demonstrated to induce acute inflammatory response and alter the balance of pro- and anti-inflammatory cells in the TME (56).

IL-23 is another cytokine that does not have an entirely clear role in tumor progression, however, in the context of immunotherapy, the association of this cytokine with CAR-T cells has also shown to be promising. MA et al. developed a strategy to couple the release and activity of IL-23 with the activation of T cells through the supplementation of T cells with the subunit IL-12β p40. The T cells that expressed p40 (p40-Td cells) produced IL-23 after activation, inducing a greater proliferation and survival of these T cells. This work demonstrated that the integration of p40 in CAR-T cells was able to increase its antitumor activity in xenograft models and syngeneic mice. This approach was also shown an acceptable safety profile, because the IL-23 produced by p40-Td cells act mainly through an autocrine mechanism with inferior effect on non-tumor cells (57).

Investigating the IL-22 functionality in a head and neck squamous cell carcinoma (HNSCC) model, a comparison of a second-generation CAR T cells with fourth-generation CAR-T cells merged with this cytokine (55) was done. *In vitro* data demonstrated that IL-22 positively regulates central memory and effective memory T cells in CD3+ T cells, resulting in higher rates of proliferation and survival of these cells. *In vivo*, the CAR-MUC1-IL-22 T cells were able to progressively decrease the size of tumors more efficiently than second-generation cells. Investigators attributed this effect to the inferior persistence of CAR-MUC1 T cells, while IL-22 can facilitate the differentiation of CAR-T cells into a TCM phenotype and improve CAR-MUC1-IL-22 T cells activity.

Chen, et al. (58) investigated a construct for the NB GD2 antigen with IL-15 (GD2.CAR.15) and without (GD2.CAR) in a xenogeneic model of neuroblastoma *in vitro* and *in vivo*. The construct with IL-15 secretion induced increased memory T cells with stem cell phenotypes, in addition to lower expression of PD-1. *In vivo*, they also tested the continue reexposure of GD2.CAR to tumor cells and the results further highlighted the role of IL-15 to promote superior survival of CAR-T cells with greater antitumor activity (58).

On your side, Lanitis et al. used a bicistronic vector with the coexpression of murine IL-15 and CAR in T cells. They observed that in non-activated IL-15 secreting CAR-T, there were low levels of IL-15 in the culture supernatant, but after antigenic stimulation there was a significant increase, proving that the secretion of IL-15 is conditioned to cell activation CAR-T by the antigen. The functionality of co-expressed IL-15 was demonstrated by increased proliferation and survival when compared to second-generation CAR-T cells, in addition to also promoting a TCM cell phenotype, inferior expression of PD-1 and superior cell activity after the antigenic challenge. They also evaluated the efficacy of 4G-CAR-T cells *in vivo* in a B16 melanoma tumor model and found that 4G-CAR-T cells showed greater persistence and greater efficacy against tumor cells when compared to CAR-T cells without IL-15 secretion (59).

Adopting another approach, Batra et al. (60) tested the cytokines IL-15 and IL-21 associated with CAR-T cells separately and together in a model of hepatocellular carcinoma (HCC). They showed that CAR-T cells that coexpress IL-15 and/or IL-21 were effective against tumor cells. CAR-T cells with the combined expression of IL-15 and IL-21 showed a less differentiated profile and longer survival in repeated exposures to tumor cells, in addition to maintaining the expression of T-cell factor-1 (TCF-1), an important factor for the development and survival of T cells. *In vivo* experiments observed that these cells (CAR-T/IL-15/IlL-21) showed a larger expansion and persistence, resulting in greater control of tumor burden and survival of animals when compared to only CAR-T cells and CAR-T cells with the secretion of isolated cytokines. These results demonstrated the superiority of the efficiency of the approach with the coexpression of IL-15 and IL-21 in the preclinical scenario, given the data needed to launch a phase I clinical trial that will recruit patients with liver tumors (NCT02932956 and NCT02905188) (60).

Recently, Duan et al. (61) working with fourth-generation CAR-T cells targeting BCMA and IL-7 and CCL19 expression (CAR-T BCMA-7×19) demonstrated that these cells had a greater capacity for expansion, differentiation, migration, and cytotoxicity. They are currently conducting a clinical trial in humans for Refractory/Recurrent Multiple Myeloma therapy using these BCMA-7×19 CAR-T cells (ClinicalTrials.gov Identifier: NCT03778346). Preliminary data of this clinical trial demonstrated that two patients receiving the treatment achieved a good response with low levels of CRS, and responses lasting more than 12 months (61).

Diverse factors impact the persistence and effectiveness of CAR T-cells, such as the presence of immunosuppressive cytokines, loss of antigen expression, T cellular exhaustion or dysfunctionality, and the variability of TME of different cancer subtypes. Cytokines functions have been seen as a great player that might give opportunities to be explored to circumvent these obstacles. In general, the association of CAR-T cells with cytokines might modulate the tumor microenvironment to privilege a pro-inflammatory profile and to recruit other cells of the immune system that traduce in superior persistence and effectiveness of CAR T cells with an acceptable safety profile (**Table 1**).

**Table 1:** Preclinical studies with TRUCKs cells.

	Cancer Types	Immune regulatory factors	Model	Findings	Summary conclusion	Reference
CD20/ Mesothelin	Mastocytoma Lung carcinoma	IL-7/CCL19	*In vitro*: P815-hCD20 cells *In vivo*: DBA/2 mice inoculated with P815-hCD20 cells	* Lower expression of immune checkpoint molecules * Induces infiltration of T cells and their interaction with DCs inside tumor tissue	* Potent anti-tumor effects and greater persistent cellular * Long-term survival in the animal model	(61)
MUC-16ecto (4H11-28z)	Ovarian cancer	IL-12	*In vitro*: SKOV3 human ovarian tumor cells *In vivo*: SCID-Beige mice with human ovarian cancer xenografts	• Robust IFNg secretion	* Enhanced antitumor efficacy * Increases survival and prolongs persistence	(50)
Glypican-3 (GPC3)	Hepatocellular carcinoma	IL15/IL21	*In vitro*: Hep3B cell line *In vivo*: NOD murine xenograft models of GPC3^+^ tumor cell	• Increase Tscm/Tn and Tcm populations	• Superior expansion, persistence, and antitumor activity	(60)
Carcino-embryonic antigen (CEA)		IL-12	*In vitro*: C15A3 cells *In vivo*: NIH-III mouse inoculated with CEA^+^ C15A3 tumor cells	• Increased numbers of macrophages and IFN-g secretion	• Greater antitumor efficacy • Effect locally restricted to the tumor lesion	(46)
Cell-surface ganglioside GD2	Neuroblastoma	IL15	*In vitro*: IMR-32, LAN-1, SKNLP cell lines *In vivo*: NSG mice - xenogeneic metastatic model of neuroblastoma (CHLA255 cell)	* Reduced expression of PD-1 and LAG-3 * Enrichment of memory and stem cell profiles	* Superior antitumor activity *in vitro* and *in vivo*	(58)
Carcino-embryonic antigen (CEA)	Pancreatic and Lung tumors	IL-12/IL-18	*In vitro*: CEA+ Panc02 cells *In vivo*: C57BL/6 mice with intra-pancreatic injection of CEA Panc02 cells	* IL-18 increased T-bet and decreased FoxO1 expression, whereas IL-12 increased both * IL-18 increase CD206 M1 macrophages and NKG2D+ NK cells, and decreased Tregs, suppressive CD103+ DCs, and M2 macrophages	* Superior activity against large pancreatic and lung tumors * Improved the overall survival	(56)
Mesothelin/ CD19	Pancreatic flank tumor Lymphoblastic leukemia	IL-18	*In vivo*: xenograft model of mesothelin-expressing pancreatic tumor AsPC1 and Nalm6 cells in NOD scid gamma (NSG) mice.	* Tendency to expand T cells to a primarily central memory (TCM) phenotype (CCR7+CD45RO+)	* Supports *in vivo* engraftment and persistence	(56)
MUC1	Head and neck squamous cell carinoma	IL-22	*In vitro*: Cal33 tumor cells *In vivo*: Mice (NOD/SCID) inoculated with HN4 cells.	* Induces MUC1 expression, with greater accuracy of CAR-T to tumor cells * Upregulate central memory and effective memory T cells	* Stronger and more effective cytotoxic	(55)
VEGFR-2	Melanoma tumors	IL15	*In vivo*: C57BL/6 mice with subcutaneous B16 melanoma tumors	* Higher proportion of naive and CM cells and fewer EM cells * Lower levels of PD-1	* CAR-T cell survival and proliferation	(58)

In summary, the TRUCKs approach demonstrates a substantial translational potential, and currently, is already being explored in clinical protocols, summarized in **Table 2**.

**Table 2:** Clinical trials with TRUCKS cells (ClinicalTrials.gov).

Title	Conditions	Status	Phase	NCT Number
Clinical Follow-up Study of CD19 CAR-T Expressing IL7 and CCL19 for Relapsed or Refractory B Cell Lymphoma	Diffuse Large B-cell Lymphoma Mantle Cell Lymphoma Transformed Follicular Lymphoma Primary Mediastinal Large B-cell Lymphoma	Completed	–	NCT04833504
Interleukin-15 and -21 Armored Glypican-3-specific Chimeric Antigen Receptor Expressed in T Cells for Pediatric Solid Tumors	Liver Cancer Rhabdomyosarcoma Malignant Rhabdoid Tumor Liposarcoma|Wilms Tumor Yolk Sac Tumor	Not yet recruiting	I	NCT04715191
huCART19-IL18 in NHL/CLL Patients	Chronic Lymphocytic Leukemia Non-hodgkin Lymphoma	Recruiting	I	NCT04684563
IL3 CAR-T Cell Therapy for Patients with CD123 Positive Relapsed and/or Refractory Acute Myeloid Leukemia	Acute Myeloid Leukemia	Not yet recruiting	Early I	NCT04599543
CD19 CAR-T Expressing IL7 and CCL19 Combined with PD1 mAb for Relapsed or Refractory Diffuse Large B Cell Lymphoma	Diffuse Large B-cell Lymphoma	Recruiting	I	NCT04381741
Interleukin-15 Armored Glypican 3-specific Chimeric Antigen Receptor Expressed in T Cells for Pediatric Solid Tumors	Liver Cancer|Rhabdomyosarcoma Malignant Rhabdoid Tumor Liposarcoma|Wilms Tumor Yolk Sac Tumor	Recruiting	I	NCT04377932
T Cells co- Expressing a Second Generation Glypican 3-specific Chimeric Antigen Receptor with Cytokines Interleukin-21 and 15 as Immunotherapy for Patients With Liver Cancer (TEGAR)	Hepatocellular Carcinoma Hepatoblastoma	Withdrawn	I	NCT04093648
Interventional Therapy Sequential with the Fourth-generation CAR-T Targeting Nectin4/FAP for Malignant Solid Tumors	Nectin4-positive Advanced Malignant Solid Tumor	Recruiting	I	NCT03932565
Integrin Î²7, BCMA, CS1, CD38 and CD138 as the Single or Compound Targets for the Fourth Generation of CAR-T Cells	RRMM	Recruiting	I	NCT03778346
Study of CAR T-Cells Targeting the GD2 With IL-15+iCaspase9 for Relapsed/Refractory Neuroblastoma or Relapsed/Refractory Osteosarcoma	Neuroblastoma|Osteosarcoma	Recruiting	I	NCT03721068
EGFR-IL12-CART Cells for Patients with Metastatic Colorectal Cancer	Metastatic Colorectal Cancer	Unknown status	I	NCT03542799
GD2 Specific CAR and Interleukin-15 Expressing Autologous NKT Cells to Treat Children with Neuroblastoma	Neuroblastoma	Recruiting	I	NCT03294954
GPC3-CAR-T Cells for Immunotherapy of Cancer with GPC3 Expression	Hepatocellular Carcinoma| Immunotherapy CARGPC3 Gene Inactivation T Cell|Squamous Cell Lung Cancer	Recruiting	I	NCT03198546
Cyclophosphamide Followed by Intravenous and Intraperitoneal Infusion of Autologous T Cells Genetically Engineered to Secrete IL-12 and to Target the MUC16 ecto Antigen in Patients with Recurrent MUC16 ecto Solid Tumors	Solid Tumors	Active, not recruiting	I	NCT02498912
Study of IFN-α Combined With CAR-T Cell Therapy in Relapsed and Refractory Acute Lymphoblastic Leukemia (R/R-ALL) B-cell Acute Lymphoblastic	B-cell Acute Lymphoblastic Leukemia	Recruiting	II	NCT04534634

## The role of cytokines in the CAR-T cells manufacturing process

**Figure 2:**
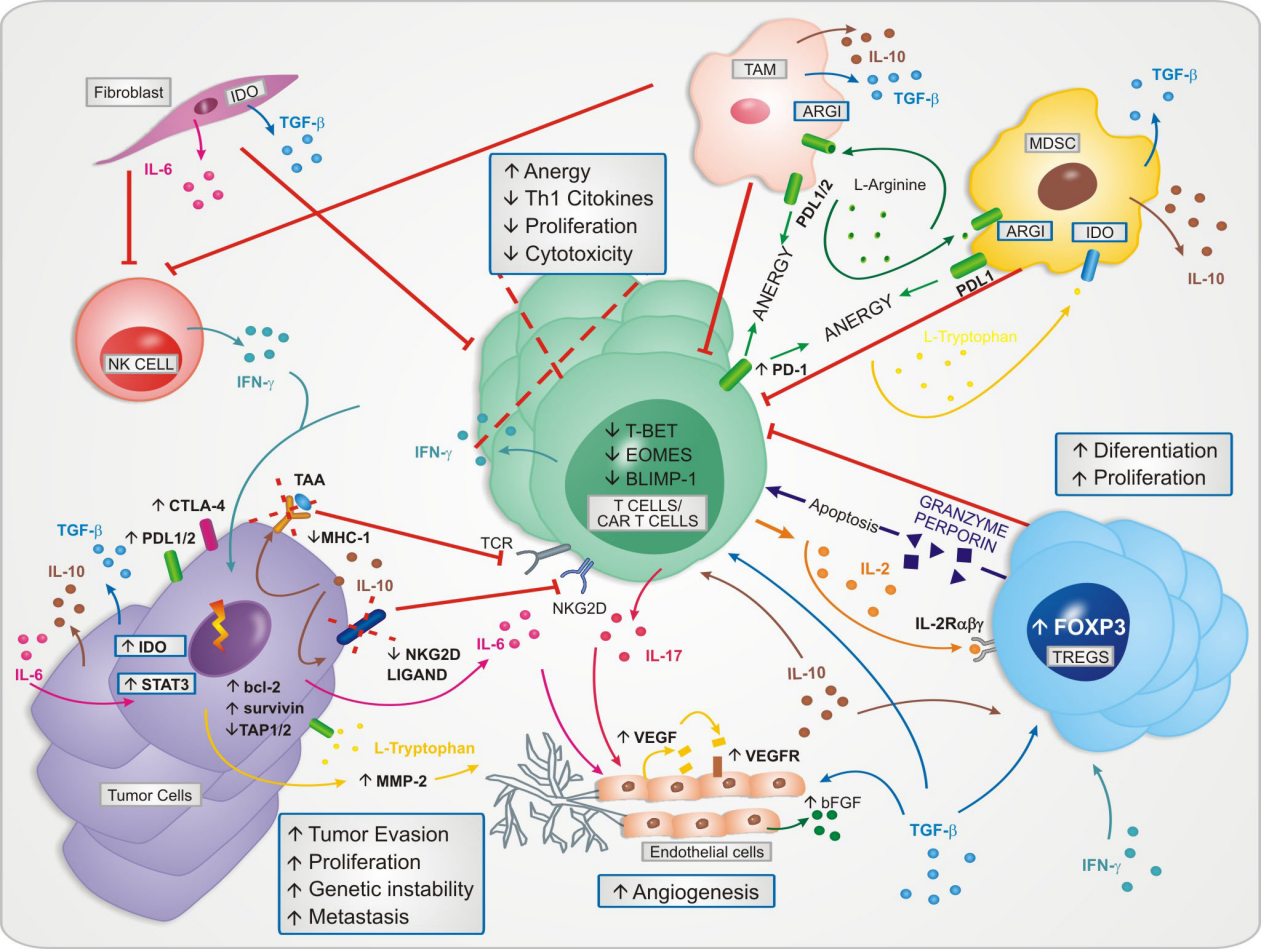
A representative scheme of CAR-T cell manufacturing platform. The definition of equipment, steps and protocols used depend on the patient, the type of tumor and clinical setting.

The CAR-T cell manufacturing platform comprises several steps where the cytokines have a central role in the quality and functionality of the final product. Firstly, the isolation and enrichment of the T cells. CD3^+^ T cells are commonly obtained from peripheral blood mononuclear cells (PBMCs), harvested from leukapheresis, and further separated by lymphocyte separation medium centrifugation by manually or automated systems. Secondly, the enrichment of specific subsets of T cells could be achieved by using magnetic microbeads such as CD3^+^, CD4^+^, CD8^+^, CD25^+^, being also used for the selection or depletion of specific T cell types within the PBMCs enabling T cell expansion and administration of the final cell product with a defined CD4:CD8 ratio (47, 62). Thirdly, the gene transfer system is normally done through viral vectors transferring the corresponding genetic information into the T cells mediating CAR expression on the T cell surface. Virus-based gene delivery systems are commonly used, and they can achieve high transduction efficiency rates. Finally, CAR-T cells are expanded by *ex vivo* culture methods and the final cell product is subjected to end-of-process formulation and cryopreservation (47, 62). Quality control testing is performed during the production as well as for the final cryopreserved product (63). These steps of CAR-T cell manufacturing are described in **Figure 2**.

Cytokines play an important role on the activation, expansion and quality of T cells for CAR-T cells manufacturing. T cells are cultured in specific mediums generally supplemented with important cytokines such as IL-2, IL-7, IL15, and IL-21, influencing the composition, quality, and phenotype of the adoptively transferred T cells. The two most used strategies for CAR-T cell production are based on either IL-2 or IL-7, with or without IL-15 (64).

As mentioned before, IL-2 drives terminal effector T cells differentiation and proliferation by upregulating perforin, granzyme B and IFN-γ and suppressing the memory cell marker, such as BCL6 and IL7RA (65, 66). IL-2 is an important cytokine to T cell culture; however, it can promote a relatively mature phenotype, with low expression of CD62L, CCR7, CD27 and CD28, which correlated with a reduced blood persistence *in vivo* (67). In addition, it is known that IL-2 favors the expansion of regulatory T cells that can inhibit the anti-tumor activity of the CAR-T cells (4, 68).

The receptors for cytokines of the γ-chain family, such as IL-7, IL-15, and IL-21, have a common CD132 or γ-chain and can reduce the CAR-T cells terminal differentiation and increase the frequency of memory stem cells, yielding improved *in vivo* persistence (69).

It was reported that supplementation of IL-15 alone can lead to reduced exhaustion marker expression, an increase of anti-apoptotic properties providing similar performance in stimulating CAR-T cell expansion, persistence *in vivo* and tumor-lysis functions *in vitro*. IL-21 is another cytokine implicated with CAR-T cells expansion memory phenotype, however, potentially correlated with inferior expansion when compared with IL-2 processing (70).

IL-7/IL-15 have shown an enhancement in activation and *ex vivo* proliferation, with inferior exhaustion markers when compared to IL-2 (84). Additionally, it was reported that a combination of IL-7/IL-15 promotes the survival and maintenance of less differentiated T cells, while IL-15 and IL-21 seem better suited for *in vivo* administration after CAR-T cell infusion and triggering differentiation in memory cells. CAR-T cells that were exposed to IL-2 and IL-15 secreted more proinflammatory cytokines and presented stronger tumor-lysis ability *in vitro* (70).

**Figure 3:**
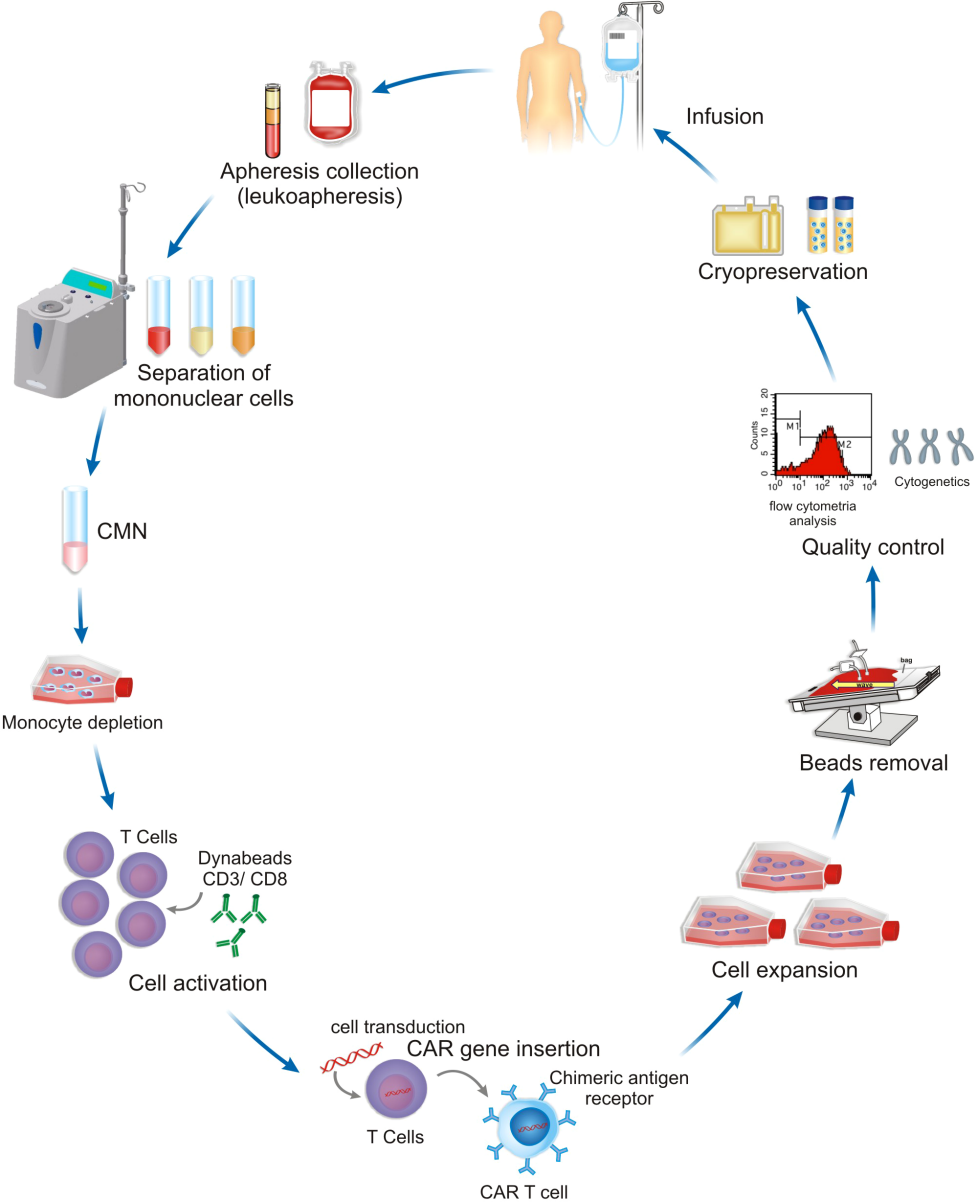
Cytokines play an important role in the generation of CD8^+^ T cells and can drive memory cell profiles.

In summary, the CAR-T cell manufacturing protocols have to be supplemented with specific cytokines during *ex vivo* production. Currently, studies mainly rely on IL-2, IL-7, IL-15, and IL-21 **Figure 3**. The optimal cytokine composition, as well as the role of other cytokines for CAR-T cell generation, is not clearly defined yet (70), mainly because it depends on the type of final product defined according to the patient, tumor, and clinical scenario.

## Cytokine release syndrome

Although the use of CAR T cells is still considered an innovative treatment, several clinical complications after infused cells have been well identified. The CRS is the most prominent side effect that is not clearly elucidated (71, 72). According to first clinical trials, up to 90% of patients treated with CAR-T will develop CRS, and half of the patients progressed to grade 3–4 or even cause death. CRS is characterized by the increase of serum cytokines levels, inflammatory markers, and generalized activation of the immune system then infusion effector cells (72–80).

Usually, CRS occurs between the first and the fourteenth day after the infusion with a wide expression of clinical manifestations. Fortunately, most patients will take a self-limited clinical course. However, few patients might require the use of anti-IL 6 specific treatment, such as tocilizumab, or high doses of steroids and the need of intensive care unit (73, 81). Initial clinical manifestation may include fever, myalgia, fatigue and might progress into hypoxia, capillary leakage, important organ dysfunction that implicate with a life-threatening condition (5, 67).

**Figure 4:**
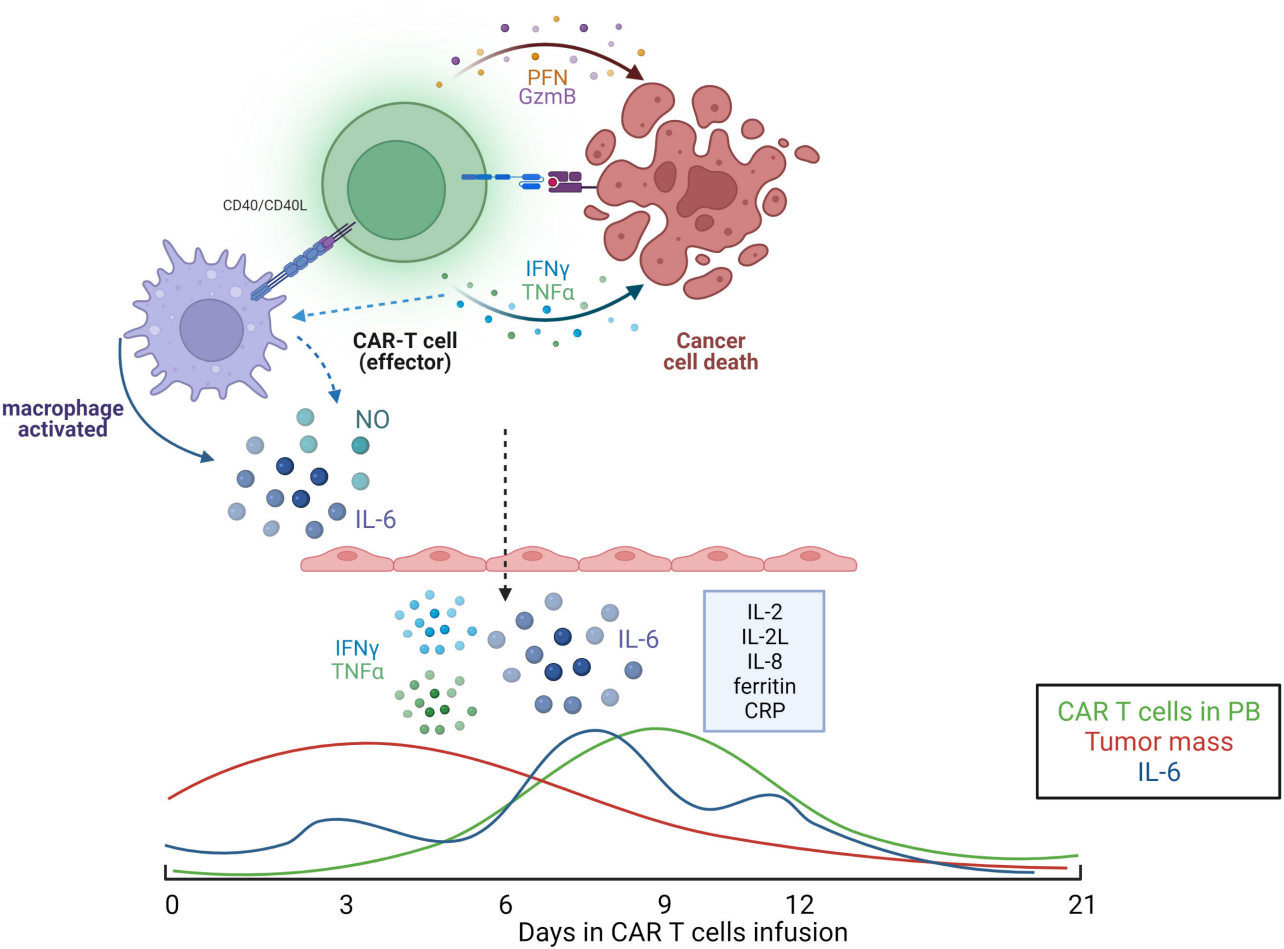
Illustrative model of cytokine secretion mechanism commonly observed in patients receiving CAR-T cell therapy. Below, kinetics of released factors involved in CRS. Illustration was created using BioRender.

CAR-T cells have an important role in the genesis of the cytokine storm, however other immunological cells are also implicated, such as macrophages and endothelial cells (67, 72–74, 82). It was observed increases in F4/80^int-lo^Ly6C^int-hi^ macrophages, which, following administration of mCD40L CD19 CAR T-cells engineered to further engage macrophages, led to markedly increased macrophage numbers, CRS symptoms, and mortality. IL-6 was predominantly produced by these macrophages, and blockade with anti-murine IL-6R antibody or with anakinra. Macrophages are also implicated with another mechanism in the genesis of CRS, the inducible nitric oxide synthase (iNOS) and nitric oxide production pathway. In addition to IL-6, other cytokines are involved in CRS such IL-1, IFN-γ, TNFα, IL-8 and IL-10, GM-SCM, also showing the importance of the cytokines in the clinical setting, as shown in the **Figure 4** (67, 72–74, 82).

Several recent studies have looked at alternative CAR T-cell strategies to improve the safety profile, such as engineering CAR T-cells with suicide genes, ON- and OFF- switches, AND/OR logic gating, or various inhibitory domains. However, these strategies directly limit CAR T-cell function (82). A possible alternative to circumvent the effects of IL-6 on CRS and preserve the antitumor activity of CAR-T cell was addressed in a study that developed a CD19 CAR T cell with a membrane-bound scFv targeting IL-6 constitutively expressed on its surface. In this approach, CAR-T cells might function as an IL-6 scavenger (83).

Another proposed mechanism by which CAR T-cells activate macrophages is direct activation through secretion of granulocyte macrophage colony-stimulating factor (GM-CSF) and GM–CSF is specifically upregulated in a CAR-dependent manner (82). This condition has led to speculation that GM–CSF may play a key role in monocyte activation, resulting in CRS association with CAR-T cell therapy. In order to reduce the release of GM-CFS, CRISPR/Cas9-mediated knockout of GM–CSF in CAR-T cells and consequently lower GM-CFS release in TME was observed in addition to preservation of the anti-tumor effect in the cell (82).

## Conclusions

CAR-T cell therapy has been recognized as a breakthrough treatment for patients with hematological malignant diseases. However, their application on the solid tumors remains limited. In this setting, cytokines have been demonstrated to have important whole related to the complex procedure that involves CAR-T cell therapy, since the preclinical development, the harvest of T cells until the infusion and clinical management. Special highlights should be done to the manufacturing process where cytokines activities have crucial impact on the quality of CAR-T cell final product.

## Author contributions

CS, AC, SC, NM, NB, FH and RG-C: wrote the manuscript. CS designed **Figure 1**. SC and TF designed **Figure 2**. FH and SC designed **Figure 3**. NM designed **Figure 4**. CS, PA, MO and RG-C reviewed the article. All authors contributed to the article and approved the submitted version.

## Funding

This article is supported by the ASH Research Global Award (2018) and the following Brazilian research agencies: CNPq (grant #442686/2020-0); CRFS has postdoctoral fellowships from FAPESP (grant# 2020/10804-3); AC (CAPES grant # 88887.597999/2021-00) and FH (CNPq grant #88887.600222/2021-00); PA and NB have CNPq technical fellowship grant#380594/2022-6 and grant# 380029/2022-7 respectively.

## Acknowledgments

We would like to thank Mrs. Sandra Navarro Bresciani for support in the design of the figures. **Figure 4** was created using BioRender.com.

## Conflict of interest

The authors declare that the research was conducted in the absence of any commercial or financial relationships that could be construed as a potential conflict of interest.

## Publisher’s note

All claims expressed in this article are solely those of the authors and do not necessarily represent those of their affiliated organizations, or those of the publisher, the editors and the reviewers. Any product that may be evaluated in this article, or claim that may be made by its manufacturer, is not guaranteed or endorsed by the publisher.
